# Annual acknowledgement of manuscript reviewers

**DOI:** 10.1186/2050-6511-14-21

**Published:** 2013-04-29

**Authors:** Christopher Morrey

**Affiliations:** 1 BioMed Central, Floor 6, 236 Gray's Inn Road, London, WC1X 8HB, UK

## Abstract

**Contributing reviewers:**

In August 2012 the BMC Series journals BMC Clinical Pharmacology and BMC Pharmacology merged to form a new title BMC Pharmacology and Toxicology. The editors of BMC Clinical Pharmacology and BMC Pharmacology would like to thank all of our reviewers who have contributed to these journals in Volume 12 (2012). The editors of BMC Pharmacology and Toxicology would also like to thank all of our reviewers who have contributed to the journal in Volume 13 (2012).

## 

Jose Adams

USA

Richard Adome

Uganda

Farzaneh Aghahosseini

Iran

Alexandra Alexopoulou

Greece

Sam Alfred

Australia

Anna Birna Almarsdóttir

Iceland

Sreedhar Amere Subbarao

India

Salvatore Amoroso

Italy

Andreas Argyriou

Greece

Yutaka Banno

Japan

Rachid Baz

USA

Josef Berger

Czech Republic

Corrado Blandizzi

Italy

David Boulton

USA

Claudio Campa

Italy

Domenico Capone

Italy

Pablo Carbonell

France

William Carson

USA

Andrea Cavalli

Italy

Kinwei Chan

USA

Zhenhai Chen

USA

Wong Chi Sang Martin

Hong Kong

Marcos Chiaratti

Brazil

Bice Chini

Italy

Cheong-Weon Cho

South Korea

Mariann Churchwell

USA

Antonio Clavenna

Italy

Pietro Cottone

USA

Joyce Cramer

USA

Cesare Cremon

Italy

Patricia De Cremoux

France

Ingrid De Meester

Belgium

Pierre De Meyts

Denmark

Edoardo De Robertis

Italy

Marco De Santis

Italy

Ellen Tveter Deilkas

Norway

Olivier Delezay

France

Shalinee Dhayal

UK

Siamak Djafarzadeh

Switzerland

Ryan Donnelly

UK

Susan Dosreis

USA

Fred Downey

USA

Heather Dowty

USA

Adrienne Einarson

Canada

Sean Ekins

USA

Elisabet Ekman

Sweden

Patrick Erah

Nigeria

Sara Erickson

USA

Cetin Erol

Turkey

Hui Feng

USA

Olavo Fernandes

Canada

Albert Figueras

Spain

Mark Fineman

USA

John Flack

USA

Louise Forsetlund

Norway

Filomena Fortinguerra

Italy

Michael France

UK

Angel-Luis García-Otín

Spain

Maria Cristina Gaudiano

Italy

Victor Gault

UK

Louis Gendron

Canada

Michael Gottesman

USA

Henk-Jan Guchelaar

Netherlands

Ezgi Gulmez

France

Philip Hansten

USA

Pirkko Härkönen

Finland

Lisa Hines

USA

Tsuneya Ikezu

USA

Kiyotoshi Inenaga

Japan

Carla Jarrett

USA

Michael Kays

USA

Terry Kenakin

USA

Attila Kertesz-Farkas

Hungary

Jong-Suk Kim

South Korea

Gil Klinger

Israel

Janet Krska

UK

Thomas Krunkosky

USA

Vidhya Kumaresan

USA

Chun Shing Kwok

UK

Anne Larson

USA

Paolo Lentini

Italy

Craig Lindsley

USA

Marie Lordkipanidze

UK

Mirte Malingre

Netherlands

Daniel Malone

USA

Michael Marino

USA

Eduardo Mariño

Spain

Stella Martin

USA

Andrew Massey

UK

Gracia Merino

Spain

Stephan Michels

Switzerland

Francis Miller

USA

Andrew Morris

USA

Jyoti Nautiyal

USA

Chiratidzo Ellen Ndhlovu

Zimbabwe

Steve Negus

USA

Charles Nhachi

UK

Alfredo Nunziata

Italy

Bello Oricha

Nigeria

Ilza Pajea

Bulgaria

Manikandan Panchatcharam

USA

Gavril Pasternak

USA

Zhiyong Peng

USA

Pablo Perez-Martinez

Spain

Itay Perlstein

USA

Wendy Qiu

USA

Emanuel Raschi

Italy

Marcia Ratner

USA

Torleif Ruud

Norway

Valentina Sabino

USA

George Sakoulas

USA

Esther Sala

Spain

Cristoforo Scavone

Brazil

Arne Schousboe

Denmark

Werner Schroth

Germany

Sam Schulman

Canada

Ganga Senarathna

Sri Lanka

Catherine Sherwin

USA

Steve Shoptaw

USA

Daniel Sitar

Canada

Angela Slitt

USA

Karl Staples

UK

Olivier Steichen

France

Mark Stein

USA

Margaret Stone

UK

Jeannine Strobl

USA

Marijo Tamburrino

USA

Giovanni Tarantino

Italy

Ilaria Tarricone

Italy

Greg Thatcher

USA

Michael Turner

Ireland

Koen Van De Wetering

Netherlands

Richard Van Rijn

USA

Mladen Vidovich

USA

Giulio Vistoli

Italy

Hidefumi Wada

Japan

Richard Wainford

USA

Clinton Webb

USA

Li-Na Wei

USA

Adrian Whitty

USA

Cara Williams

USA

Collynn Woeller

USA

Qingyou Xia

China

Jialin Xu

USA

Hidenori Yamasue

Japan

Osamu Yasuda

Japan

Laxman Yetukuri

Finland

Giacomo Zoppini

Italy

## Pre-publication history

The pre-publication history for this paper can be accessed here:

http://www.biomedcentral.com/2050-6511/14/21/prepub

